# Supplementary material for: DMRforPairs: identifying Differentially Methylated Regions between unique samples using array based methylation profiles
Source: BMC Bioinformatics. 2014 May 15;15:141. doi: 10.1186/1471-2105-15-141 (PMC4046028; doi:10.1186/1471-2105-15-141)
Supplement: Additional file 2 — DMRforPairs output for the comparison of A431-MCF7 and NA17018-NA17105. Please start from the HTML files in each folder. Available via the BMC Bioinformatics website. [file 1471-2105-15-141-S2.zip › 1394847754114233_MOESM2_ESM/NA17105M_NA17018F/figures/33681.pdf]

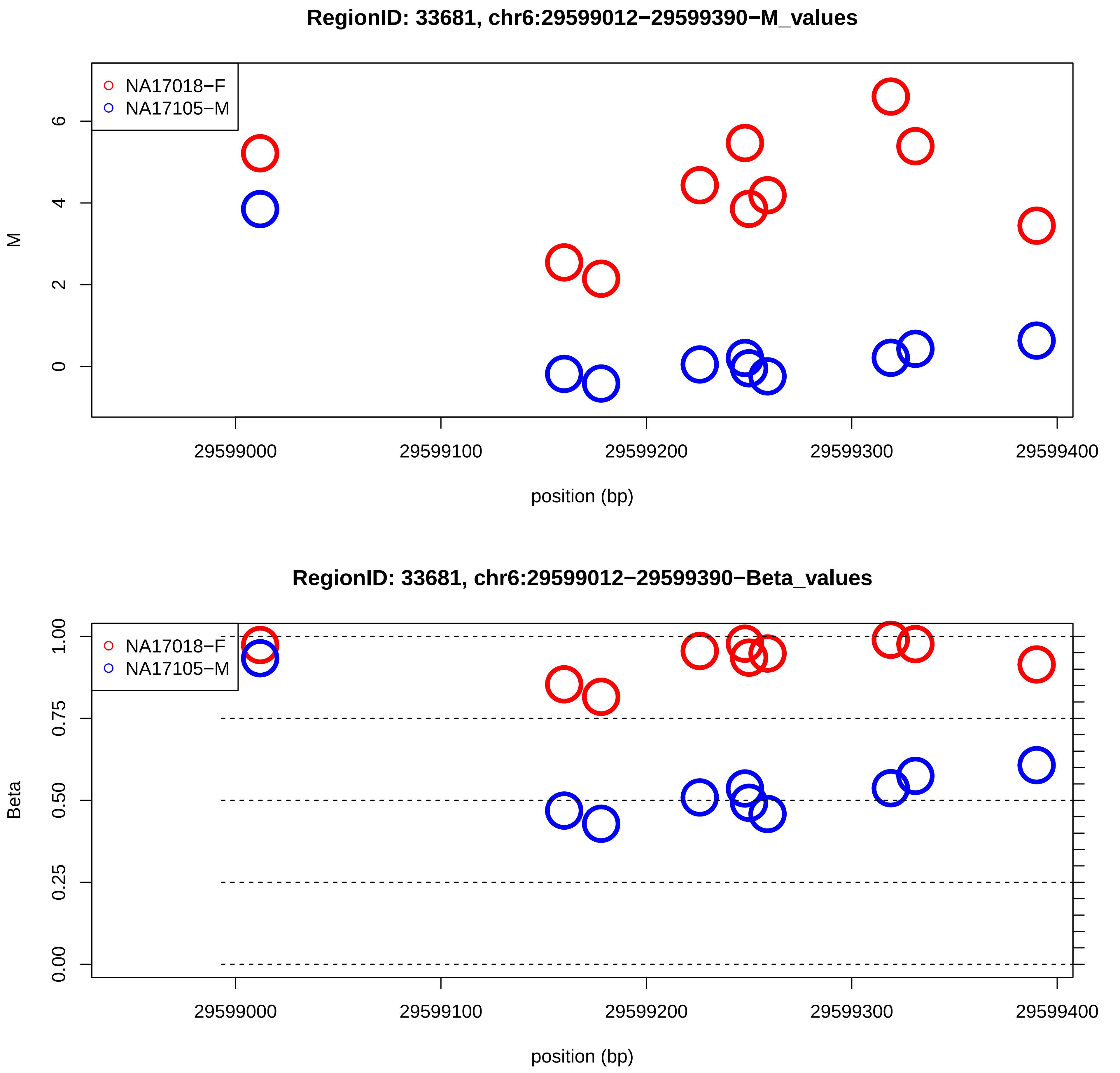

RegionID: 33681, chr6:29599012−29599390−M_values
NA17018−F
NA17105−M
M
0
2
4
6
29599000
29599100
29599200
29599300
29599400
position (bp)
RegionID: 33681, chr6:29599012−29599390−Beta_values
NA17018−F
NA17105−M
Beta
0.00
0.25
0.50
0.75
1.00
29599000
29599100
29599200
29599300
29599400
position (bp)

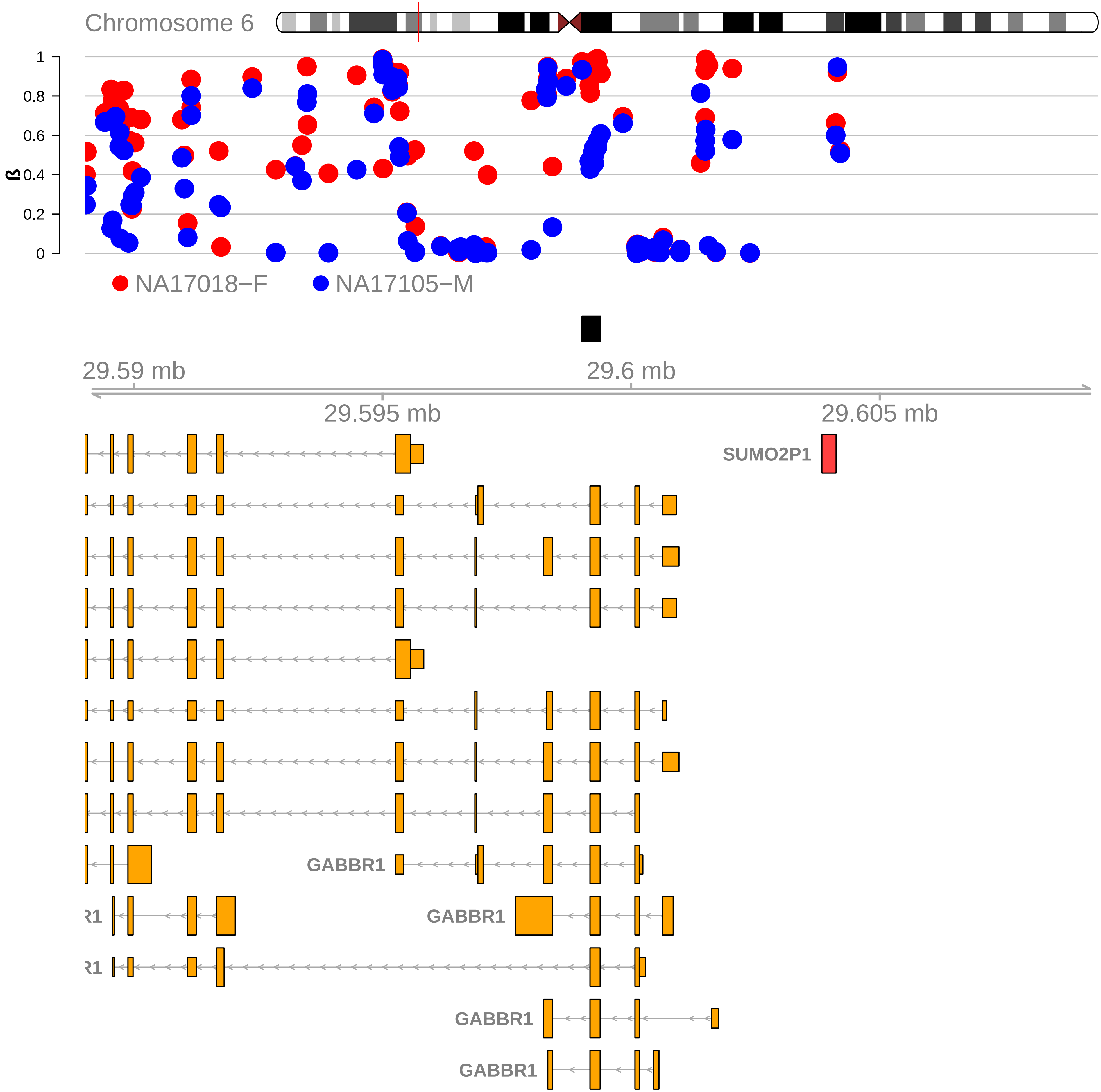

Chromosome 6
ß
1
0.8
0.6
0.4
0.2
0
NA17018−F
NA17105−M
29.59 mb
29.6 mb
29.595 mb
29.605 mb
SUMO2P1
GABBR1
GABBR1
GABBR1
GABBR1
